# Inflammatory mediator bradykinin increases population of sensory neurons expressing functional T-type Ca^2+^ channels

**DOI:** 10.1016/j.bbrc.2016.02.118

**Published:** 2016-04-29

**Authors:** Dongyang Huang, Ce Liang, Fan Zhang, Hongchao Men, Xiaona Du, Nikita Gamper, Hailin Zhang

**Affiliations:** a Department of Pharmacology, Hebei Medical University, Shijiazhuang, PR China; b School of Biomedical Sciences, Faculty of Biological Sciences, University of Leeds, Leeds, UK

**Keywords:** T-type Ca^2+^ channels, Inflammation, Bradykinin, P2Y receptors, Prostaglandin, Nociceptor, BK, Bradykinin, DRG, Dorsal root ganglion, LVA, Low voltage activated Ca^2+^ channels, NE, Norepinephrine, PGE2, Prostaglandin E_2_, PLC, Phospholipase C, TRPV1, Transient receptor potential cation channel subfamily V member 1

## Abstract

T-type Ca^2+^ channels are important regulators of peripheral sensory neuron excitability. Accordingly, T-type Ca^2+^ currents are often increased in various pathological pain conditions, such as inflammation or nerve injury. Here we investigated effects of inflammation on functional expression of T-type Ca^2+^ channels in small-diameter cultured dorsal root ganglion (DRG) neurons. We found that overnight treatment of DRG cultures with a cocktail of inflammatory mediators bradykinin (BK), adenosine triphosphate (ATP), norepinephrine (NE) and prostaglandin E_2_ (PGE2) strongly increased the population size of the small-diameter neurons displaying low-voltage activated (LVA, T-type) Ca^2+^ currents while having no effect on the peak LVA current amplitude. When applied individually, BK and ATP also increased the population size of LVA-positive neurons while NE and PGE2 had no effect. The PLC inhibitor U-73122 and B_2_ receptor antagonist, Hoe-140, both abolished the increase of the population of LVA-positive DRG neurons. Inflammatory treatment did not affect Ca_V_3.2 mRNA or protein levels in DRG cultures. Furthermore, an ubiquitination inhibitor, MG132, did not increase the population of LVA-positive neurons. Our data suggest that inflammatory mediators BK and ATP increase the abundance of LVA-positive DRG neurons in total neuronal population by stimulating the recruitment of a ‘reserve pool’ of Ca_V_3.2 channels, particularly in neurons that do not display measurable LVA currents under control conditions.

## Highlights

• Inflammatory mediators bradykinin and ATP increase the population of DRG neurons displaying T-type Ca^2+^ currents.
• This effect is mediated by the phospholipase C signaling cascade.
• Increase in the proportion is not correlated with the increased expression or decreased degradation of the CaV3.2 channel.

## 1 Introduction

T-type Ca^2+^ channels are increasingly recognized as an important ion channel in peripheral pain pathways [1], [2], [3], [4]. The family consists of three subunits, Ca_V_3.1–Ca_V_3.3, encoded by CACNA1G, CACNA1H and CACNA1I genes [5]. These channels have fast kinetics and low (−70 to −60 mV) threshold for activation, enabling them to be partially active at voltages near the neuronal resting membrane potential. Due to the negative activation threshold T-type Ca^2+^ currents are often called ‘low voltage-activated’ (LVA) currents. In the peripheral somatosensory system, T-type Ca^2+^ channels are expressed in small, TRPV1-positive nociceptive neurons [6], [7], [8] and in two populations of low-threshold mechanoreceptors (LTMRs): Aδ- and C-LTMRs, which innervate skin hair follicles [8], [9], [10]. Ca_V_3.2 is the predominant T-type channel isoform expressed in sensory neurons [11]. Ca_V_3.2 expression was reported in various compartments of peripheral fibers including peripheral nociceptive nerve endings and axons of skin afferents [7], [9], nodes of Ranvier of Aδ fibers [9] and presynaptic terminals of nociceptive fibers in the spinal cord ([12] but *cf.* [9]). Conditional knock-out of Ca_V_3.2 [9] or specific knock-down of this subunit in dorsal root ganglion (DRG), using intrathecal injection of antisense oligonucleotides [13], [14], resulted in potent anti-nociceptive effects in models of neuropathic and inflammatory pain. Pharmacological inhibitors of T-type Ca^2+^ channels consistently display analgesic efficacy in rodent pain models [15], [16], [17]. Ca_V_3s are clinically validated drug targets for pain [2] and several novel selective T-type channel blockers are currently under clinical trials as analgesics [18], [19], [20].

A flip side of Ca_V_3.2 expression in pain pathways is that an increase in this channel activity and/or abundance can have a pro-algesic effect. LVA Ca^2+^ currents are often increased in pathological pain conditions, such as diabetic neuropathy [14], [21], peripheral nerve injury or inflammation [22], [23], [24]. Mechanistically, an enhancement of channel trafficking to the plasma membrane (perhaps via the N-linked glycosylation) [25], [26] and enhanced retention at the plasma membrane due to the increased deubiquitination [23] were reported as potential contributors to the increased abundance of LVA channels in these pain conditions. In the present study we investigated the effect of inflammatory conditions on functional expression of Ca_V_3.2 in small-diameter DRG neurons.

## 2 Materials and methods

**DRG culture**. DRG neurons were cultured as described [27], [28]. Adult Sprague Dawley rats (170 g–180 g) were humanely euthanized by isoflurane overdose in accordance with the guidelines of the Animal Care and Ethical Committee of Hebei Medical University, Shijiazhuang, China. DRGs from all spinal levels were extracted and dissociated using collagenase/dispase method as described [27], [28]. Dissociated cells we cultured in DMEN supplemented with GlutaMax I, 10% fetal calf serum, penicillin (50 U/ml) and streptomycin (50 μg/ml) and plated on poly-d-lysine coasted glass coverslips for 2–5 days in a humidified incubator (37 °C, 5% CO_2_).

**Electrophysiology**. All recordings were made at room temperature using Multiclamp 700B amplifier in combination with pCLAMP 10.4 software (Axon Instruments, USA). A whole-cell configuration of the patch clamp technique was used throughout. Patch pipettes were pulled from borosilicate glass using a horizontal micropipette puller (P-97, Sutter Instruments, USA) and fire-polished to a final resistance of 2–4 MΩ. The standard intracellular solution contained (in mM): 135 CsCl; 3 MgCl_2_; 10 EGTA; 10 HEPES; 3 Mg-ATP; 0.6 GTP (pH 7.4 adjusted with CsOH). The standard bath solution contained (in mM): 150 TEA-Cl; 2 CaCl_2_; 10 HEPES; 10 glucose (pH 7.4 adjusted with CsOH). LVA currents were measured by 50 ms square voltage pulses to −40 mV from a holding potential of −90 mV. Recordings were sampled at 4 kHz. A low-profile perfusion chamber fed by a gravity perfusion system was used for solution exchange.

**Live cell imaging**. DRG were dissociated as described above and cultured on 15 mm glass-bottom dishes (NEST). Twenty four hours after dissociation the dishes were put into a humidified (37 °C, 5% CO_2_) live cell imaging station (GSI-D35, TOKAI HIT) and monitored for the next 24 h under control conditions or in the presence of inflammatory mediators. Images were taken every 1 min. At the end of the 24 h incubation, the number of morphologically intact neurons was evaluated and compared to that at the beginning of incubation.

**Immunohistochemistry**. DRG were dissociated as described previously and cultured on 10 mm cover-glasses in 24-well plates in the presence and absence of the test compounds. Cover-glasses with DRG cultures were washed with 0.1 M phosphate buffered saline (PBS; Sigma) and blocked for 2 h with blocking buffer (10% Goat serum in 0.1 M PBS; Sigma). Primary anti-Ca_V_3.2 antibody (Alomone, Rabbit, Cat#: ACC-025) was diluted (1:200) in 0.3% Triton X-100/PBS buffer and incubated overnight at 4 °C. On a following day sections were washed with 0.1 M PBS and incubated with secondary antibody (Jackson, FITC-Goat Anti-Rabbit IgG) for 4 h at room temperature. Each cover-glass was washed with PBS and incubated with DAPI for 10 min, followed by repeated PBS washes and mounted on microscope slides using Vectashield. Staining was visualized using a confocal fluorescent microscope (Leica, SP-5).

**RT-PCR**. DRG were dissociated and cultured on 10 mm cover-glasses in 24-well plates in the presence or absence of the test compounds. Total RNA was extracted using a commercial RNA isolation kit (RNAiso, Takara). Isolated RNA was dissolved in 20 μl DEPC-treated water and reverse-transcribed using an RT reagent kit (PrimeScript™ with gDNA Eraser, Takara) and a thermal cycler (Mastercycler, Eppendorf). qPCR reactions were performed using a kit (SYBR Premix Ex TaqII (Tli RNase H Plus), Takara) and the fluorescent DNA detected and quantified with a FQD-48A(A4) system (BIOER). The following primers were used: Cacna1h sense: 5′-TGCCCACGGAGTCTATGAGT-3′; Cacna1h antisense: 5′-GTTGTAGGGGTTCCGGATGT-3′ and Gapdh sense: 5′-GACATGCCGCCTGGAGAAAC-3′; Gapdh antisense: 5′-AGCCCAGGATGCCCTTTAGT-3′.

**Chemicals**. NE, Hoe-140, MG132 and A-317491 were purchase from MCE; PGE2 was from TCI; all other chemicals were from Sigma.

**Statistics**. All data are given as mean ± S.E.M. Differences between groups were assessed by Student's t-test (paired or unpaired, as appropriate). χ^2^ test was used to determine whether there were differences in the proportion of cells responding to a treatment. The differences were considered significant at P ≤ 0.05. Statistical analyses were performed using Origin 8.6 (OriginLab Corporation, Northampton, CA, USA).

## 3 Results

**Fig. 1:**
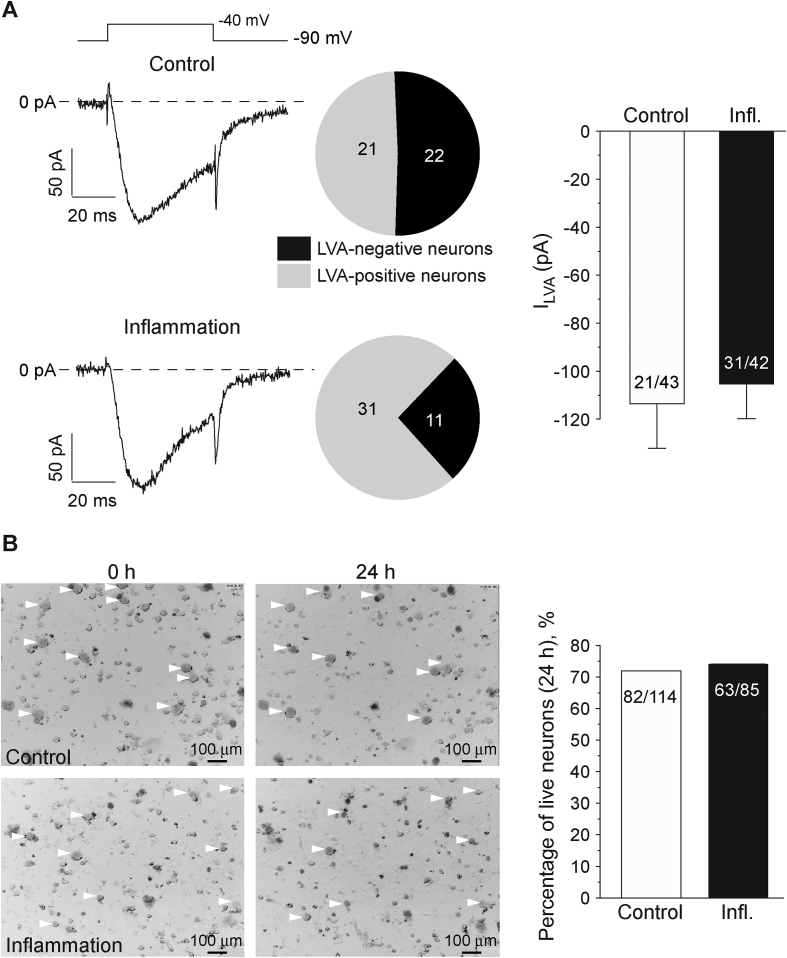
Inflammatory treatment increases pool size of DRG neurons displaying low-voltage activated (LVA) Ca^2+^ currents. (A) Cocktail of inflammatory mediators (100 nM BK, 2 μM ATP, 500 nM NE, 500 nM PGE2) increased percentage of LVA-positive neurons. Shown are exemplary current traces from control (top) and inflammatory cocktail-treated small-diameter DRG neurons recorded with whole-cell patch clamp using voltage protocol depicted above. Pie-charts summarize the percentage of LVA-positive neurons. Bar chart on the right summarizes the LVA current amplitudes in LVA-positive neurons in both conditions. (B) Exemplary micrographs of live cell imaging at 0 h (left) and 24 h (right) with or without inflammatory treatment (see Methods for detail). Bar chart on the right summarizes the percentage of morphologically intact neurons at the end of 24 h incubation in either condition. For (A) and (B) number of neurons is indicated within the charts; data from at least three independent preparations.

Up-regulation of activity [29] or abundance [23], [24] of T-type Ca^2+^ channels was reported to contribute to pain and hyperalgesia observed in several inflammatory models, including experimentally-induced irritable bowel syndrome [24] and complete Freund's adjuvant (CFA)-induced chronic inflammation [23], [29]. Thus, we tested if and how functional expression of LVA channels is changed under the inflammatory conditions *in vitro*. We cultured DRG neurons overnight (24 h) in the presence of a cocktail of inflammatory mediators (100 nM BK; 2 μM ATP; 500 nM NE and 500 nM PGE2) to mimic inflammation, and performed patch-clamp recording to evaluate LVA Ca^2+^ current amplitude and also the population size of the DRG neurons expressing LVA currents (‘LVA-positive neurons’). We recorded from small-diameter (∼20 μm diameter, ∼25 pF capacitance) DRG neurons, which are predominantly TRPV1-positive under our experimental conditions [27]. Overnight treatment with the inflammatory cocktail significantly increased the percentage of the LVA-positive neurons from 21/43 (48.8%) to 31/42 (73.8%; p < 0.05; Fig. 1A) but did not affect the peak LVA current amplitude (Fig. 1A). The mean LVA current amplitudes were −113.6 ± 18.5 pA (n = 21) and −105.3 ± 14.5 pA (n = 31) in control and inflammatory treated neurons, respectively. There was no significant effect of the inflammatory cocktail on the LVA current voltage dependence or kinetics (not shown).

One possible explanation for the observed results could be that the inflammatory treatment causes neuronal death, and that LVA-negative neurons are more susceptible to this effect for some unknown reason. However, during 24 h live cell imaging of DRG cultures we did not observe any significant cell death induced by the inflammatory cocktail, as compared with control (Fig. 1B). After 24 h incubation 82/114 (71.9%) neurons monitored in control culture and 63/85 (74.1%) neurons monitored in the inflammatory cocktail-treated culture remained morphologically intact (Fig. 1B). Thus, a feasible explanation for an increase in the proportion of LVA-positive neurons after the inflammatory treatment is the increase of functional T-type channel abundance in neurons that do not normally display measurable LVA currents.

**Fig. 2:**
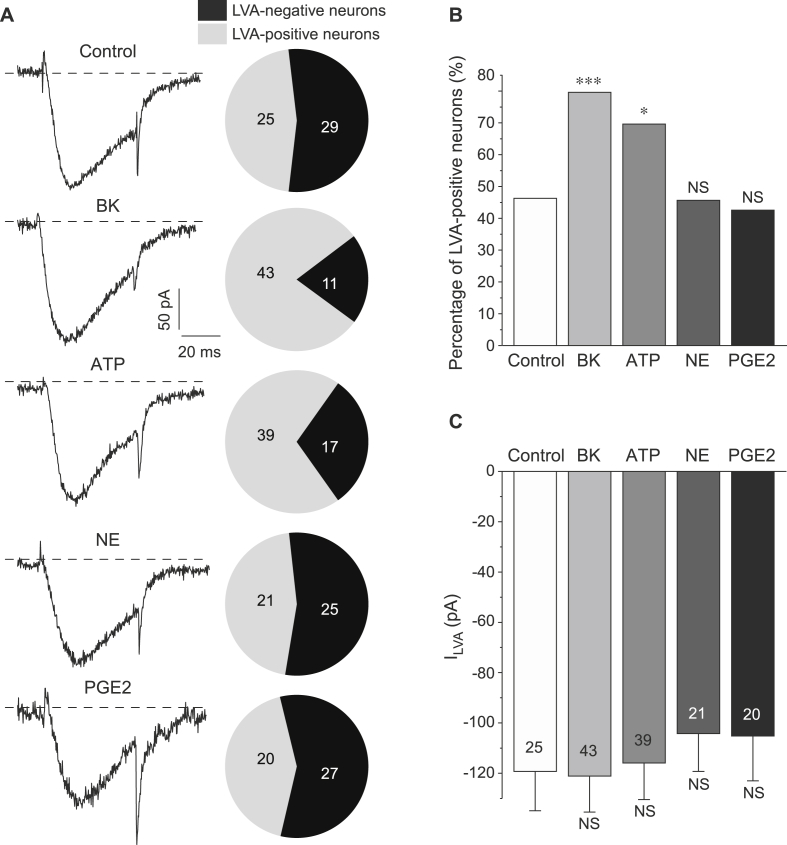
BK and ATP but not NE or PGE2 increase the proportion of LVA-positive DRG neurons. (A) Exemplary current traces (left) and pie-charts summarizing the percentage of LVA-positive neurons (right) for DRG neurons individually treated with 100 nM BK, 2 μM ATP, 500 nM NE, 500 nM PGE2 or vehicle control (as indicated). (B) Statistical comparison of the proportion of LVA-positive neurons for each condition tested in (A); *, *** significantly different from the control group with P < 0.05 or P < 0.001; χ^2^ test. (C) Summary of the LVA current amplitudes in LVA-positive neurons for each condition tested in (A). For (A–C) number of neurons is indicated within the charts; data from at least three independent preparations.

We next tested the effects of individual inflammatory mediators on both the LVA amplitude and the incidence of LVA-positive neurons in the DRG culture. Overnight incubation with BK (100 nM) or ATP (2 μM) significantly increased the percentage of LVA-positive neurons from 25/54 (46.3%; control) to 43/54 (79.6%; BK; p < 0.05) or 39/56 (69.6%; ATP; p < 0.05), respectively (Fig. 2A–B). Again, there was no effect on the peak LVA Ca^2+^ current amplitude by either treatment (Fig. 2A, C). Neither NE (500 nM) nor PGE2 (500 nM) had an effect on the percentage of LVA-positive neurons: 21/46 (45.6%) in NE-treated group and 20/47 (42.6%) in PGE2-treated group were LVA-positive (Fig. 2A, B; p > 0.05 compared to control). Both compounds had no effect on LVA Ca^2+^ current amplitude (Fig. 2A, C).

**Fig. 3:**
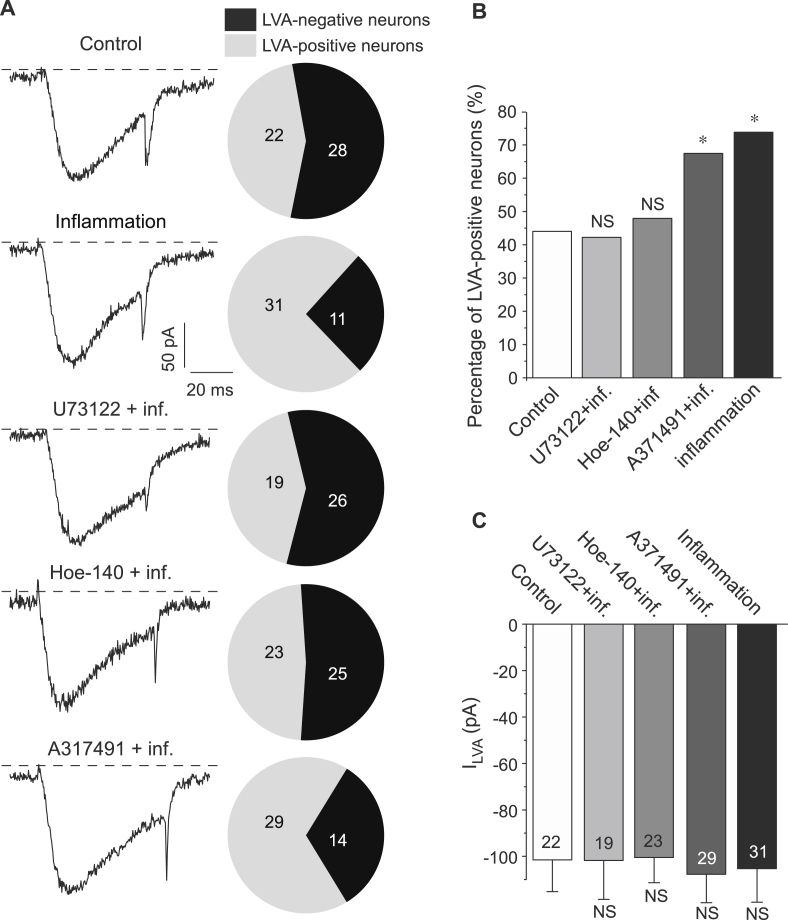
The increase in the proportion of LVA-positive DRG neurons is mediated by the G_q/11_-PLC signaling cascade. (A) Exemplary current traces (left) and pie-charts summarizing the percentage of LVA-positive neurons (right) for DRG cultures treated with (i) the cocktail of inflammatory mediators (100 nM BK, 2 μM ATP, 500 nM NE, 500 nM PGE2), (ii) the same cocktail with the addition of either PLC inhibitor U73122 (1 μM), or (iii) B_2_ receptor antagonist Hoe-140 (10 nM) or (iv) P2X_2/3_ antagonist A317941 (1 μM), as indicated. Also shown is the exemplary trace from the saline-treated control neuron. (B) Statistical comparison of the proportion of LVA-positive neurons for each condition tested in (A); * significantly different from the control group; P < 0.05; χ^2^ test. (C) Summary of the LVA current amplitudes in LVA-positive neurons for each condition tested in (A). For (A–C) number of neurons is indicated within the charts; data from at least three independent preparations.

We next tested the signaling cascade underlying the BK and ATP effects on the pool size of LVA-positive DRG neurons. BK signals through the constitutive B_2_ receptors and inducible B_1_ receptors, both belong to the G_q/11_-coupled G protein coupled receptors [30]. ATP activates ionotropic P2X receptors (DRG neurons express predominantly P2X_2_, P2X_3_ and their multimers [31], [32]) and metabotropic P2Y receptors (DRG express predominantly P2Y_1_ and P2Y_2_ [33]). Similarly to BK receptors [30], P2Y_1_ and P2Y_2_ are coupled to the G_q/11_ signaling cascade which involves activation of phospholipase C (PLC), hydrolysis of membrane phosphoinositide phosphatidylinositol 4,5-bisphosphate (PIP_2_) into inositol trisphosphate (IP_3_) and diacylglycerol (DAG) and triggering the appropriate downstream signaling cascades [30], [34]. PLC inhibitor U-73122 (1 μM) completely abolished the effect of the inflammatory cocktail on the abundance of LVA-positive neurons in DRG culture. Thus, in the cultures treated overnight with the inflammatory cocktail in the presence of U-73122 only 19/45 (42.2%) of neurons displayed LVA currents, a proportion similar to that observed in the control conditions (22/50, 44.0%; p < 0.05; Fig. 3A, B). There was no effect of U-73122 on the peak LVA Ca^2+^ current amplitude (Fig. 3C).

We next used a specific antagonist of B_2_ receptors, Hoe-140, to confirm the contribution of the BK signaling cascade to the action of the inflammatory cocktail. In cultures treated with the inflammatory cocktail in the presence of Hoe-140 (10 nM) overnight only 23/48 (48.0%) of neurons displayed LVA currents, a proportion similar to that in the control conditions (Fig. 3A, B). Consistent with the effect of the PLC inhibitor U-73122, P2X_2_/P2X_3_ receptor antagonist A-317491 (1 μM) did not abolish the increase in the proportion of LVA-positive neurons induced by the inflammatory treatment: there were 29/43 (67.5%) LVA-positive neurons in cultures treated with the inflammatory cocktail and A-317491, thus producing a similar proportion to cultures treated with the inflammatory cocktail only (31/42 or 73.8% neurons) (Fig. 3A, B). Neither Hoe-140 nor A-317491 produced an effect on the LVA current amplitude (Fig. 3A, C).

**Fig. 4:**
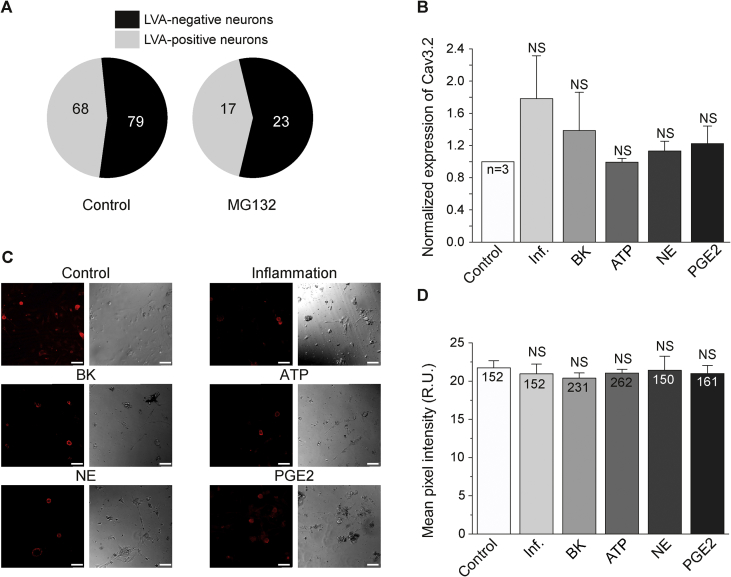
Expression of Ca_V_3.2 T-type channel subunit in DRG neurons is not affected by the inflammatory treatment. (A) Pie-charts of the proportion of LVA-positive neurons in cultures incubated for 24 h with (right) or without (left) 5 μM MG132. Control group represents pooled data from all the control cultures used in this study. (B) Summary of mRNA levels in DRG cultures incubated for 24 h with either a vehicle control or the cocktail of inflammatory mediators (100 nM BK, 2 μM ATP, 500 nM NE, 500 nM PGE2) or each of these mediators individually at the specified concentration. (C) Exemplary confocal images of DRG neurons treated in the same way as in (B) (scale bar is 50 μM) and stained with anti-Ca_V_3.2 antibody. (D) Summary of Ca_V_3.2 staining intensity for conditions shown in (C). For (A–D) number of neurons is indicated within the charts; data from at least three independent preparations.

Earlier studies suggest that at least two mechanisms may contribute to the increased abundance of T-type channels in nociceptors under inflammatory conditions: (i) increased trafficking [25], [26] and (ii) enhanced membrane retention due to the increased deubiquitination by the deubiquitinating enzyme USP5 [23]. In addition, increased expression can also potentially contribute. To investigate these mechanisms we first tested the effect of the proteasome inhibitor, MG132, on the pool size of LVA-positive neurons in DRG culture. MG132 reduces the degradation of ubiquitin-conjugated proteins; it was successfully used to prevent degradation of Ca_V_3.2 channels in DRG neurons [23]. MG132 (5 μM) treatment had no effect on the proportion of LVA-positive neurons. After overnight treatment with MG132 there were 17/40 (42.5%) of LVA-positive neurons (Fig. 4A). MG132 did not affect LVA current amplitude (−124.3 ± 23.5 pA, n = 17), which is consistent with the previous finding [23]. We also tested the effect of inflammatory conditions on the expression of Ca_V_3.2 in DRG neurons using RT-PCR and immunostaining. There was no significant increase of Ca_V_3.2 transcript in DRG cultures after overnight incubation in the presence of the inflammatory cocktail (Fig. 4B). Confocal imaging of cultured DRG neurons immunostained for Ca_V_3.2 revealed there were also no significant changes in the total Ca_V_3.2 protein abundance in DRG neurons after either of inflammatory treatments (inflammatory cocktail or individual inflammatory mediators; Fig. 4C, D).

## 4 Discussion

Here we demonstrate that inflammatory mediators, acting via the G_q/11_-PLC signaling cascade, strongly increase the pool size of the LVA-positive DRG neurons. Among the mediators tested (BK, ATP, NE and PGE2) only BK and ATP demonstrated such an activity when used individually. BK perhaps plays a dominant role in the effect since selective B_2_ receptor antagonist abolished the action of the inflammatory cocktail (although some cross-reactivity of Hoe-140 at P2Y receptors cannot be excluded). We could not detect an increase in the expression of Ca_V_3.2 after the inflammatory treatment on mRNA or protein levels. Similarly, the inability of MG132 to increase the proportion of LVA-positive neurons argues against reduced ubiquitination as the underlying reason for the effect. It seems logical to propose that stimulation of G_q/11_-PLC signaling pathway by B_2_ or P2Y receptors stimulates recruitment of the ‘reserve pool’ of Ca_V_3.2 channels, particularly in neurons which do not normally display robust LVA currents. Thus, some neurons that do not normally express functional Ca_V_3.2 at the plasma membrane may possess an intracellular store of channels, and BK treatment may promote their membrane insertion, making these neurons *de novo* LVA-positive. This hypothesis may explain a paradoxical lack of effect of the inflammatory treatment on the mean LVA Ca^2+^ current amplitude. There might be an effective control mechanism responsible for the tonic levels of functional Ca_V_3.2 channels at the plasma membrane and while inflammatory treatment does not change this level, it triggers the recruitment of a ‘Ca_V_3.2 reserve’ in some ‘nominally’ LVA-negative neurons, converting these into the LVA-positive. A very similar phenomenon was recently reported for delta-opioid receptors (DOR); indeed BK treatment potently increased the pool size of the DOR-competent DRG neurons without enhancing overall DOR activity in the individual neurons [35]. Taken together our data report novel mechanism that may contribute to the inflammatory overexcitability of peripheral somatosensory fibers.
